# The Torque Teno Virus Titer in Saliva Reflects the Level of Circulating CD4^+^ T Lymphocytes and HIV in Individuals Undergoing Antiretroviral Maintenance Therapy

**DOI:** 10.3389/fmed.2021.809312

**Published:** 2022-01-14

**Authors:** Layla Honorato, Steven S. Witkin, Maria Cássia Mendes-Correa, Ana Luiza Castro Conde Toscano, Iara Moreno Linhares, Anderson Vicente de Paula, Heuder Gustavo Oliveira Paião, Vanessa Salete de Paula, Amanda de Oliveira Lopes, Silvia Helena Lima, Vanessa de Cássia Raymundi, Noely Evangelista Ferreira, Almir Ribeiro da Silva Junior, Karim Yaqub Abrahim, Paulo Henrique Braz-Silva, Tania Regina Tozetto-Mendoza

**Affiliations:** ^1^Laboratory of Virology (LIM 52), Department of Infectious Diseases, Instituto de Medicina Tropical de São Paulo, Universidade de São Paulo, São Paulo, Brazil; ^2^Department of Obstetrics and Gynecology, Weill Cornell Medicine, New York, NY, United States; ^3^Hospital Dia, Instituto de Infectologia Emílio Ribas, São Paulo, Brazil; ^4^Departamento de Ginecologia e Obstetrícia, Universidade de São Paulo, São Paulo, Brazil; ^5^Laboratory of Molecular Virology, Instituto Oswaldo Cruz, Fundação Oswaldo Cruz, Rio de Janeiro, Brazil; ^6^Faculdade de Odontologia da Universidade de São Paulo, São Paulo, Brazil

**Keywords:** CD4+ lymphocytes, HIV, Kaposi's Sarcoma, saliva, torque teno virus, anellovirus

## Abstract

**Introduction:**

Torque teno virus (TTV) is a non-pathogenic virus present in body fluids. Its titer in the circulation increases in association with immune suppression, such as in HIV-infected individuals. We evaluated if the TTV titer in saliva from HIV-positive individuals undergoing antiretroviral therapy (ART) was related to the circulating CD4+ T lymphocyte concentration and the HIV titer.

**Methods:**

Saliva was collected from 276 asymptomatic individuals undergoing ART, and an additional 48 individuals positive for AIDS-associated Kaposi's Sarcoma (AIDS-KS). The salivary TTV titer was measured by gene amplification analysis. The circulating CD4+ T lymphocyte and HIV levels were obtained by chart review.

**Results:**

TTV was detectable in saliva from 80% of the asymptomatic subjects and 87% of those with AIDS-KS. In the asymptomatic group the median log_10_ TTV titer/ml was 3.3 in 200 males vs. 2.4 in 76 females (*p* < 0.0001). TTV titer/ml was 3.7 when HIV was acquired by intravenous drug usage, 3.2 when by sexual acquisition and 2.4 when blood transfusion acquired. The salivary TTV titer was inversely correlated with the circulating CD4+ T lymphocyte level (*p* < 0.0001) and positively correlated with the circulating HIV concentration (*p* = 0.0005). The median salivary TTV titer and circulating HIV titer were higher, and the CD4+ count was lower, in individuals positive for AIDS-KS than in the asymptomatic subjects (*p* < 0.0001).

**Conclusion:**

The TTV titer in saliva is a potential biomarker for monitoring immune status in individuals undergoing ART.

## Introduction

Torque teno virus (TTV), first described in 1997, is a non-enveloped DNA virus (1) belonging to the *Anelloviridae* family and the *Alphatorquetenovirus* genus (2). There are at least 29 major TTV species that exhibit a high degree of genomic heterogeneity (1–4). TTV is recognized as a commensal non-pathogenic virus with a worldwide distribution. It is ubiquitously detected in body fluids in up to 90% of healthy individuals (2, 4, 5). It has been suggested that the TTV level in saliva may be elevated as compared to its level in the circulation, peripheral blood mononuclear cells or in body sites such as in liver or bone marrow (6–9). Therefore, the utility of salivary TTV determination may be of clinical value for evaluating immune status.

A higher prevalence of TTV and a wider range of TTV genotypes has been reported to be present in the circulation of HIV-infected individuals as compared to uninfected healthy people (10, 11). In addition, there appears to be an inverse correlation between the CD4+ T lymphocyte count and the plasma level of TTV in the context of HIV infection (10, 12, 13).

The aim of the present study was to investigate the presence and titer of TTV in saliva samples from asymptomatic HIV-positive individuals undergoing anti-retroviral therapy (ART), as well as in AIDS patients being treated for Kaposi's Sarcoma (AIDS-KS), and to determine if this analysis could serve as a potential biomarker of immune status, in addition to the circulating CD4+ T lymphocyte and HIV level.

## Materials and Methods

### Subjects and Study Sites

The current protocol is a component of our previous studies on HIV-infected patients with and without Kaposi sarcoma (CAAE: 55771116.0.1001.0065) (14, 15). Saliva samples from HIV-infected subjects undergoing ART were collected as part of a 2008 study of molecular characterization of herpesvirus human type 8 (HHV-8), the causal agent of Kaposi's sarcoma. However, saliva samples from the AIDS-KS patients were scarce, all consumed and necessitated collection of new salivary samples from AIDS-KS patients between 2016 and 2018. The primary subjects in this cross-sectional study were 276 asymptomatic and HIV-positive who were all outpatients at the Hospital das Clínicas, of the Faculdade de Medicina of the Universidade de São Paulo, São Paulo, Brazil. An additional 48 saliva samples were collected from subjects with AIDS-KS at Dia Hospital of the Instituto de Infectologia Emílio Ribas (IIER), São Paulo, Brazil. All clinical information as well as each subject's circulating HIV and CD4+ T lymphocyte concentration, were obtained by chart review. The HIV level was determined by a standard real time polymerase chain reaction with an analytical sensitivity of 50 copies/ml; the circulating CD4+ concentration was measured by routine Fluorescence Activated Cell Sorting (FACS) analysis. All subjects were taking combination ART. The chemotherapy (CT) regime for AIDS-KS was based on the available regimen (IIER), ABV, Daunorubicin or Doxorubicin, alternatively.

### Saliva Collection and Molecular Analysis of TTV

Saliva was obtained within 30 days of blood collection for CD4+ and HIV analysis. The saliva collection protocol was as described previously (16, 17) and a 2 ml sample was cryopreserved at-−80°C until used. Nucleic acid was extracted and purified by using a DNA extraction kit (Real Genomics Biotech Corp.), according to the manufacturer's instructions. The quality of the total DNA was monitored by PCR amplification of the human β-globin sequence (18), prior to investigation of viral DNA. All samples were suitable for viral DNA amplification as judged by the results of this internal control.

The primers and probe sequences for DNA TTV amplification were as described previously: Forward primer 5′-GTGCCGIAGGTGAGTTTA-3′; Reverse primer 5′-AGCCCGGCCAGTCC-3′; Probe: FAM5′- TCAAGGGGCAATTCGGGCT-3′ (3, 5). For real time quantitative PCR reaction (qPCR), a standard curve was generated, as described previously, with known amounts of the synthetic oligonucleotide: 5′TTCGT AGCCCGGCCAGTCC CGTAT AGCCCGAATTGCCCCTTGA ATGCGT TAAACTCACCTTCGGCAC CTGATA−3′ (16, 19). The qPCR mix was prepared with forward and reverse primers at 250 nM and the probe at 62 nM and a standard input of ~100 ng of DNA template per reaction in 12.5 μl of 2X TaqMan™ Universal Master Mix (Thermo Fisher Scientific, Warrington, UK). Thermocycling conditions consisted of two initial heat activation steps of 50°C for 2 min and 95°C for 15 s, followed by 50 cycles of 15 s at 95°C and 1 min at 60°C, in a Quantstudio™ 5 instrument. The data were analyzed using QuantStudio Design & Analysis Software v.1.4.1. The limit of sensitivity of TTV qPCR was 40 copies/ml at a >95% detection rate (16, 19).

### Statistical Analysis

The Mann-Whitney test or the Kruskal-Wallis test were used to compare results between groups, as appropriate. Associations between the TTV titer and the CD4 or HIV concentration were analyzed by the Spearman rank correlation test. A *p*-value < 0.05 was considered significant. The GraphPad Prism 9 software (San Diego, CA) was used for all analyses.

### Ethical Approval

The Institutional Review Board committees at Hospital das Clínicas da Faculdade de Medicina da Universidade de São Paulo CAPPESP 0290/07 and Emilio Ribas CC No. 48/2016 previously approved the project (CAAE: 55771116.0.1001.0065). All subjects provided written informed consent before their participation and saliva collection. An amendment to waive the informed consent for the current protocol on salivary TTV titer and immune status was approved by the same committees (5.054.321 and 5.065.362) in October, 2021.

## Results

### Characteristics of the Asymptomatic Subjects and Relation to TTV Titer in Saliva

The mean age of the study subjects was 44.8 +/−9.2 years and usage of ART was for 4.0 +/−0.9 years. Characteristics of the asymptomatic group and the relationship to the salivary TTV titer is shown in Table 1. The majority of the subjects were male (72.5%) and their median log_10_ TTV titer/ml (3.3) was higher than in the female subjects (2.4) (*p* < 0.0001, Mann-Whitney test). The racial distribution was 60.5% White and 39.5% non-White and there was no association between race and TTV titer. The mode of HIV acquisition was 42.4% in men having sex with men, 27.9% from blood transfusion, 16.7% by heterosexual sex and 13.0% by intravenous (IV) drug ingestion. The median log_10_ TTV titer/ml was different in each group (*p* < 0.0001, Kruskal-Wallis test), being highest in the IV drug users (3.7) and lowest in the transfusion group (2.4). However, there were no differences in the CD4 or HIV levels by source of infection (*p* > 0.05, Kruskal-Wallis test).

**Table 1:** Association between characteristics of study subjects and TTV in saliva.

**Parameter**	**N**°**subjects**	**Median Log_**10**_ TTV/ml (interquartile range)**
Male	200	3.3 (2.3, 4.3)^a^
Female	76	2.4 (<0.3, 3.5)
White	167	3.2 (1.0, 4.0)
Non-white	109	2.9 (1.7, 4.3)
**HIV acquisition**		
Sexual acquisition^b^	164	3.2 (2.3, 4.2)
IV drug use	36	3.7 (2.4, 4.4)
Blood transfusion	77	2.4 (<0.3, 3.6)

a *p < 0.0001 vs. female (Mann-Whitney test);*

b *p < 0.0001 for mode of HIV acquisition (Kruskal-Wallis test)*.

### Association Between TTV in Saliva and Circulating CD4+lymphocytes

The salivary TTV titer was inversely related to the circulating CD4+ T lymphocyte concentration (*p* < 0.0001, Spearman rank correlation test). The log_10_ TTV titer/ml at different CD4+ concentrations is shown in Table 2. The TTV titer decreased from 5.7 when the CD4+ level was <200 cells/mm^3^, to 3.7 when CD4+ was between 201 and 350 cells/mm^3^, to 3.4 when the level was between 351 and 500 cells/mm^3^, to 2.9 when CD4+ was >500 cells/mm^3^ (*p* < 0.0001 Kruskal-Wallis test).

**Table 2:** Association between CD4+ cell count and TTV in saliva.

**CD4+ (mm^**3**^)**	**N**°**subjects**	**Median Log_**10**_ TTV/ml (interquartile range)^**a**^**
<200	13	5.7 (3.9, 6.6)
201–350	35	3.7 (2.4, 5.3)
351–500	64	3.4 (1.0, 4.2)
>500	164	2.9 (1.7, 3.8)

a *p < 0.0001 (Kruskal-Wallis test)*.

### Association Between TTV in Saliva and HIV in the Circulation

HIV was below the level of detection (<50 particles/ml) in 189 (68.5%) of the subjects. The association between log_10_ HIV titer in the circulation and the log_10_ TTV titer in saliva is shown in Table 3. The salivary TTV level increased in proportion to the circulating HIV level. The median log_10_ TTV titer/ml was 2.9 when HIV was not detected, 3.5 when the log_10_ HIV titer/ml was between 1.8–3.0 and 3.8 when it was between 3.1–4.0 and 4.1 when the log_10_ HIV level/ml was >4.0 (*p* = 0.0005, Kruskal-Wallis test). As expected, the HIV titer was negatively correlated with the CD4+ concentration (*p* < 0.0001, Spearman rank correlation test).

**Table 3:** Association between TTV in saliva and HIV in the circulation.

**Median Log_**10**_ HIV/ml**	**N**°**subjects**	**Median Log_**10**_ TTV/ml (interquartile range)^**a**^**
Undetectable	191	2.9 (1.6, 3.8)
1.8–3.0	34	3.5 (2.2, 4.6)
3.1–4.0	18	3.8 (2.6, 4.6)
>4.0	33	4.1 (2.6, 5.2)

a *p = 0.0005 (Kruskal-Wallis test)*.

### TTV in Individuals With AIDS-KS

The 47 subjects with AIDS-KS were younger than the asymptomatic individuals (38.2 vs. 44.8 years, *p* < 0.0001), had a lower median log_10_ CD4+ level (127 vs. 559 mm^3^, *p* < 0.0001) and a higher circulating median log_10_ HIV titer/ml (2.4 vs. <1.0, *p* < 0.0001). The median log_10_ TTV titer/ml in saliva was also greatly elevated in the AIDS-KS group (5.3) as compared to the asymptomatic group (3.1) (*p* < 0.0001). The time using ART was similar in the AIDS-KS and asymptomatic groups (4.6 vs. 4.0 years, respectively). All analyses were by the Mann-Whitney test. Similar to findings in the asymptomatic group, the circulating CD4+ concentration was inversely related to the salivary TTV titer in the group with AIDS-KS (*p* < 0.0001, Spearman rank correlation test). Regardless of the CT regimen or ART combination, the TTV was inversely proportional to the CD4+ count in 40 patients with AIDS-KS receiving CT (Supplementary Table 1, Description of characteristics of AIDS-KS subjects).

### TTV and HIV Titers in Relation to the CD4+ Lymphocyte Count

**Figure 1:**
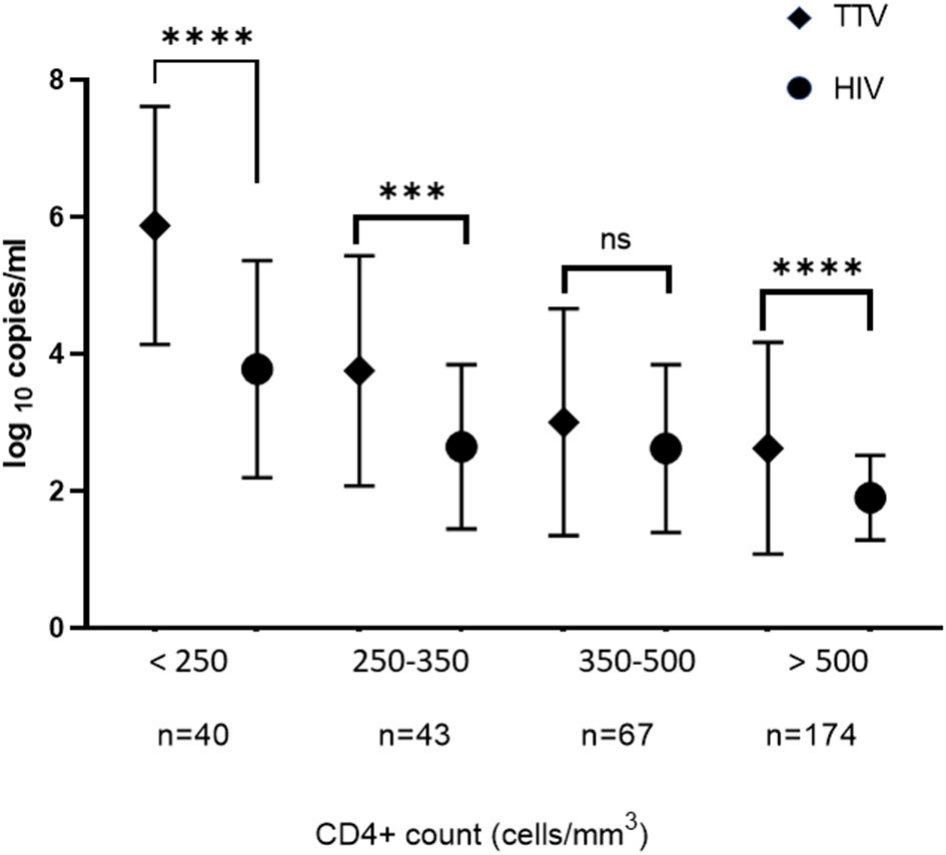
Means of titers of TTV in the saliva and HIV in the circulation in relation to the CD4+ lymphocytes count from all subjects being treated with antiretroviral therapy. The TTV and HIV titers were proportionately increased when CD4+ count was decreased. The vertical bar indicates the Standard Deviation value of the titer of TTV and HIV in log_10_ copies/ml. ****p < 0.0001; ***p < 0.0005; ns, statistically not significant.

An inverse relation between the CD4+ level and titers of both TTV/HIV was also observed in this study (Figure 1), demonstrating a suitable sensitivity for TTV analysis in the saliva. The TTV titer in saliva was higher than the HIV titer in the circulation in relation to the CD4 + level of <250, 250–350 and >500 cells/mm^3^ (^****^p < 0.0001, Mann Whitney test).

## Discussion

In our study of HIV-infected individuals who were currently on ART, the majority had detectable levels of TTV in their saliva. In both asymptomatic subjects and those positive for AIDS-KS, the TTV level was significantly elevated in parallel to the concentration of HIV in the circulation and significantly reduced as the concentration of CD4+ lymphocytes increased in the circulation. These fluctuations in the TTV level strongly suggest that analysis of the salivary TTV level is a sensitive biomarker for CD4+ status and HIV production in HIV-positive individuals currently undergoing ART. Our findings are consistent with earlier studies that demonstrated an association between the extent of TTV viremia in the systemic circulation and immune dysfunction in HIV-positive individuals (12, 13, 20–22). The analysis of TTV titers in bone marrow and spleen tissues also confirmed that elevations in TTV parallel AIDS progression (22). Serial analyses of TTV concentrations in plasma from individuals living with HIV further reinforces the inverse correlation between TTV titer and CD4 count (10, 12, 13, 20). Our observation of elevated salivary TTV levels in HIV-positive subjects with AIDS-KS as compared to asymptomatic HIV-positive individuals is also consistent with this association.

Investigations of the rate of TTV detection in blood from HIV-positive individuals have varied from <50% to >90% (13, 20, 21, 23). This discordance is likely due in large part to the choice of primers utilized for TTV detection (11, 13, 24, 25). For the quantitation of the TTV titer in saliva in our study we utilized primers and probes specific for the untranslated regions of the TTV genome in a protocol yielding an analytical sensitivity (LOD >95%) of 40 copies/ml (5, 16). The resulting detection of TTV in saliva from 80% of asymptomatic HIV-positive individuals supports the high sensitivity of our analysis.

The salivary TTV titer in women in our study was lower than in men. This is consistent with reports of higher circulating TTV levels in men than in women (26, 27). Differences in hormone levels between men and women have been implicated in influencing TTV titers and responses to HIV infection (28, 29).

The salivary TTV titer in our study subjects was highest in individuals who acquired HIV from IV drug use and lowest in those with transfusion-acquired HIV. A study of TTV in blood from individuals infected with HIV also reported the highest levels in IV drug users (11). There were no differences in the CD4 or HIV levels by source of infection in the asymptomatic individuals in our study. We propose that the TTV titer in saliva will vary with the level of physiological stress and immune suppression, and that this may very well differ between individuals with different modes of HIV acquisition. For example, IV drug users are at increased risk to acquire other infectious diseases as well as multiple TTV genotypes. However, we agree that this interpretation requires direct support and so the reasons for the observed disparity in TTV titer remains to be determined. Possibly, additional immune-related variables other than the CD4+ T lymphocyte level are responsible for the observed variations in TTV titer.

Study limitations should be acknowledged. Subjects' blood samples were not available to us for TTV analysis and thus we are unable to comment on the relative titers of TTV in saliva and in blood. Our study also only analyzed salivary TTV at a single time point. Sequential analyses might have led to a more accurate correlation between TTV abundance in saliva and variation in T cell immune responses. Patient knowledge and recall of the event responsible for HIV acquisition may be incomplete or faulty. Lastly, the absence of information on the possible influence of ART or CT on the TTV titer remains to be determined.

In conclusion, our findings suggest that the analysis of TTV titers in saliva may be of value as an additional and sensitive tool for monitoring the immune status in HIV-infected patients on ART. Similarly, a salivary TTV assay may also be beneficial as a non-invasive procedure to model the level of immunosuppression in other infectious or non-infectious disorders.

## Data Availability Statement

The original contributions presented in the study are included in the article/Supplementary Material, further inquiries can be directed to the corresponding author/s.

## Ethics Statement

The studies involving human participants were reviewed and approved by Ethics and Research Committee local CAPPEsq (number 1.560.798) and Emilio Ribas (CC No 48/2016). CAAE: 55771116.0.1001.0065, parecer number 5.054.321 and 5.065.362. The patients/participants provided their written informed consent to participate in this study.

## Author Contributions

TT-M, LH, SW, and MM-C: conceptualization. LH, AC, VP, AL, HP, SL, VR, NF, AS, and PB-S: methodology. TT-M, SW, MM-C, IL, and PB-S: validation. TT-M, LH, and SW: formal analysis. AC, LH, TT-M, KA, HP, VP, SL, VR, AL, NF, AS, and PB-S: investigation. TT-M and LH: resources. TT-M, KA, LH, SW, IL, MM-C, and PB-S: data curation. TT-M, LH, AC, and SW: writing—original draft preparation. SW: writing—review and editing. SW and LH: visualization. TT-M: supervision. TT-M and MM-C: project administration and funding acquisition. All authors contributed to the article and approved the submitted version.

## Funding

The research was supported by CNPq (Conselho Nacional de Desenvolvimento Cientifico e Tecnológico), an agency of the Brazilian Ministry of Science and Technology (Grants - 423401/2018-1- Universal Project Modality). Additional financial support was provided by the Laboratory of Virology (LIM52) of Department of Infectious Diseases of Hospital das Clínicas of the Faculdade de Medicina of the Universidade de São Paulo.

## Conflict of Interest

The authors declare that the research was conducted in the absence of any commercial or financial relationships that could be construed as a potential conflict of interest.

## Publisher's Note

All claims expressed in this article are solely those of the authors and do not necessarily represent those of their affiliated organizations, or those of the publisher, the editors and the reviewers. Any product that may be evaluated in this article, or claim that may be made by its manufacturer, is not guaranteed or endorsed by the publisher.
