# Supplementary material for: The Torque Teno Virus Titer in Saliva Reflects the Level of Circulating CD4+ T Lymphocytes and HIV in Individuals Undergoing Antiretroviral Maintenance Therapy
Source: Front Med (Lausanne). 2022 Jan 14;8:809312. doi: 10.3389/fmed.2021.809312 (PMC8795607; doi:10.3389/fmed.2021.809312)
Supplement: Supplementary file 1 [file Table_1.DOCX]

**Supplementary Information**

**Table S1. Description of characteristics of AIDS-KS subjects**

|  |  | **Patients** | | |  | |  | **Viral load (Log_10_/ copies ml)** | |  | **Treatment** | |
| --- | --- | --- | --- | --- | --- | --- | --- | --- | --- | --- | --- | --- |
|  |  |  |  |  |  |  |  |  |  |  |  |  |
| **No** | **Gender** | | **Age (years)** | **CD4 count /mm3** | | **HIV acquisition** |  | **HIV** | **TTV** |  | **ART** | **Chemotherapy** |
| **1** | M | | 18 | 538 | | Sexual acquisition |  | 1,70 | 1,88 |  | TDF + 3TC + EFZ | Liposomal Doxorubicin |
| **2** | M | | 45 | 741 | | Sexual acquisition |  | 1,70 | 2,39 |  | AZT + 3TC + LPV/r | Liposomal Doxorubicin |
| **3** | M | | 34 | 145 | | Sexual acquisition |  | 5,30 | 5,34 |  | TDF + 3TC + EFZ | None |
| **4** | M | | 43 | 118 | | Sexual acquisition |  | 1,70 | 4,08 |  | EFZ + 3TC + EFZ | Liposomal Doxorubicin |
| **5** | M | | 43 | 289 | | Sexual acquisition |  | 3,00 | 1,78 |  | TDF + 3TC + ATV | ABV / Liposomal Doxorubicin |
| **6** | M | | 21 | 39 | | Sexual acquisition |  | 2,17 | 1,44 |  | 3TC + TDF + ATV | ABV |
| **7** | M | | 46 | 242 | | Sexual acquisition |  | 2,34 | 3,11 |  | 3TC + TDF + EFZ | ABV |
| **8** | M | | 41 | 237 | | Sexual acquisition |  | 1,72 | 4,63 |  | TNF + 3TC + LPV/r | Liposomal Doxorubicin |
| **9** | M | | 51 | 370 | | Sexual acquisition |  | 1,70 | 5,46 |  | AZT + 3TC + EFZ | ABV / Liposomal Doxorubicin |
| **10** | M | | 39 | 290 | | Sexual acquisition |  | 2,51 | 4,99 |  | TDF + 3TC + EFZ | ABV / Liposomal Doxorubicin |
| **11** | M | | 32 | 12 | | Sexual acquisition |  | 4,72 | 5,65 |  | AZT + 3TC + LPV | ABV / Liposomal Doxorubicin |
| **12** | F | | 51 | 136 | | Sexual acquisition |  | 5,16 | 5,62 |  | TDF + 3TC + EFZ | None |
| **13** | M | | 25 | 65 | | Sexual acquisition |  | 3,88 | 6,10 |  | ATV + RTV + TDF + 3TC | None |
| **14** | M | | 33 | 542 | | Sexual acquisition |  | 1,70 | 0,00 |  | 3TC + TDF + EFZ | Liposomal Doxorubicin |
| **15** | M | | 28 | 106 | | Sexual acquisition |  | 3,69 | 2,47 |  | EFZ + 3TC + TNF | ABV |
| **16** | M | | 25 | 6 | | Sexual acquisition |  | 1,73 | 7,15 |  | 3TC + AZT + LPV/r | Liposomal Doxorubicin |
| **17** | M | | 50 | 916 | | NI |  | 1,70 | 3,93 |  | AZT + 3TC + IDV | ABV |
| **18** | M | | 41 | 220 | | NI |  | 1,70 | 4,76 |  | TDF + 3TC + EFV | None |
| **19** | M | | 22 | 103 | | NI |  | 5,39 | 7,59 |  | 3TC + TDF + EFV | ABV |
| **20** | M | | 33 | 329 | | Sexual acquisition |  | 4,04 | 4,90 |  | 3TC + TDF + EFZ | ABV |
| **21** | M | | 28 | 35 | | IV drug use |  | 3,29 | 7,19 |  | ABC + 3TC + LPV/r | None |
| **22** | M | | 24 | 79 | | Sexual acquisition |  | 5,49 | 6,90 |  | TDF + 3TC +RTV | None |
| **23** | M | | 41 | 669 | | Sexual acquisition |  | 1,70 | 1,55 |  | AZT +3TC + LPV/r | ABV / Daunoxome |
| **24** | M | | 64 | 667 | | NI |  | 1,70 | 0,00 |  | d4T + 3TC + EFV | ABV / Daunoxome |
| **25** | M | | 53 | 891 | | NI |  | 1,70 | 0,83 |  | AZT + 3TC | ABV / Daunoxome |
| **26** | M | | 34 | 4 | | NI |  | 5,34 | 7,77 |  | 3TC + TDF + EFZ | ABV |
| **27** | M | | 21 | 23 | | Sexual acquisition |  | 4,60 | 6,60 |  | TDF + 3TC + EFZ | ABV |
| **28** | M | | 33 | 7 | | NI |  | 2,11 | 6,36 |  | TDF + 3TC + ATV | None |
| **29** | M | | 50 | 117 | | Sexual acquisition |  | 5,09 | 5,31 |  | 3TC + TDF + EFV | ABV |
| **30** | M | | 38 | 29 | | Sexual acquisition |  | 3,27 | 8,11 |  | 3TC + TDF + EFV | None |
| **31** | M | | 31 | 94 | | Sexual acquisition |  | 4,60 | 5,34 |  | 3TC + TDF + ATV/r | ABV |
| **32** | F | | 33 | 115 | | Sexual acquisition |  | 6,09 | 6,07 |  | TDF + 3TC + DRV | ABV |
| **33** | M | | 48 | 328 | | Sexual acquisition |  | 1,70 | 2,56 |  | 3TC + AZT + LPV/r | ABV |
| **34** | M | | 48 | 32 | | IV drug use |  | 1,70 | 6,81 |  | 3TC + TDF + ATV | ABV |
| **35** | M | | 36 | 533 | | NI |  | 4,70 | 2,59 |  | 3TC + TDF + EFV | ABV |
| **36** | M | | 42 | 98 | | Sexual acquisition |  | 2,81 | 7,20 |  | 3TC + TDF + EFZ | ABV |
| **37** | M | | 41 | 15 | | Sexual acquisition |  | 5,99 | 7,03 |  | TDF + 3TC + EFZ | ABV |
| **38** | M | | 49 | 100 | | Sexual acquisition |  | 1,70 | 5,68 |  | 3TC + TDF + EFZ | ABV |
| **39** | M | | 28 | 273 | | Sexual acquisition |  | 3,29 | 4,20 |  | 3TC + TDF + EFV | ABV |
| **40** | M | | 50 | 69 | | NI |  | 2,35 | 6,56 |  | 3TC + TDF + DTG | ABV |
| **41** | M | | 33 | 19 | | NI |  | 6,33 | 7,46 |  | 3TC + TDF + EFZ | ABV |
| **42** | F | | 47 | 373 | | Sexual acquisition |  | 1,70 | 1,70 |  | d4T + 3TC + ATZ + RTV | Liposomal Doxorubicin |
| **43** | M | | 22 | 4 | | Sexual acquisition |  | 5,16 | 7,46 |  | ABC + 3TC + DTG | ABV |
| **44** | M | | 51 | 36 | | IV drug use |  | 5,71 | 7,75 |  | TDF + 3TC + DTG | ABV |
| **45** | M | | 33 | 357 | | Sexual acquisition |  | 1,70 | 4,41 |  | 3TC + TDF + EFV | ABV |
| **46** | M | | 35 | 191 | | NI |  | 1,70 | 4,23 |  | ABC + 3TC + RAL | ABV |
| **47** | M | | 64 | 588 | | Sexual acquisition |  | 2,08 | 1,41 |  | NVP + ATV + 3TC | Liposomal Doxorubicin |
| **48** | M | | 33 | 648 | | Sexual acquisition |  | 1,90 | 6,62 |  | NI | Liposomal Doxorubicin |
|  |  | |  |  | |  |  |  |  |  |  |  |

NI - Not informed

**List of medical abbreviations**

**TDF –** Tenofovir Disoproxil Fumarate **3TC -** Lamivudine
**EFZ/EFV -** Efavirenz **AZT -** Azidothymidine
**LPV/r** – Lopinavir/ritonavir
**TNF** – Tumor Necrosis Factor **RTV** - Ritonavir
**IDV** - Indinavir **ABC** - Abacavir **d4T** - Stavudine **ATV/r** – Atazanavir/ritonavir
**DRV** - Darunavir
**DTG** - Dolutegravir **NVP** – Nevirapine
**ABV** – Adriamycin, Bleomycin, Vincristine
